# Oil Essential Mouthwashes Antibacterial Activity against *Aggregatibacter actinomycetemcomitans*: A Comparison between Antibiofilm and Antiplanktonic Effects

**DOI:** 10.1155/2013/164267

**Published:** 2013-05-18

**Authors:** Matteo Erriu, Francesca Maria Giovanna Pili, Enrica Tuveri, Daniela Pigliacampo, Alessandra Scano, Caterina Montaldo, Vincenzo Piras, Gloria Denotti, Andrea Pilloni, Valentino Garau, Germano Orrù

**Affiliations:** ^1^Dipartimento di Chirurgia e Scienze Odontostomatologiche, Università di Cagliari, 09121 Cagliari, Italy; ^2^Department of Dentistry and Maxillofacial Surgery, Section of Periodontics, Sapienza University of Rome, 00161 Rome, Italy

## Abstract

The aim of this work is to determine the antibacterial activity of three marketed mouthwashes on suspended and sessile states of *Aggregatibacter actinomycetemcomitans*. The efficacy of two commonly used products in clinical practice, containing essential oils as active ingredients (menthol, thymol, methyl salicylate, and eucalyptol) in association with or without alcohol, has been evaluated in comparison with a chlorhexidine-based mouthwash. The microtiter plate assay, in order to obtain a spectrophotometric measurement of bacterial responses at growing dilutions of each antiseptic, was used for the study. The analysis revealed that a good antibacterial activity is reached when the abovementioned mouthwashes were used at concentration over a 1/24 dilution and after an exposure time of 30 seconds at least. In conclusion, the alcoholic mouthwash appears to have a better biofilm inhibition than its antiplanktonic activity while the nonalcoholic product demonstrates an opposite effect with a better antiplanktonic behavior.

## 1. Introduction

*Aggregatibacter actinomycetemcomitans* (*Aa*) is a gram-negative coccobacillus, facultative anaerobe, closely associated with the aetiology of severe periodontitis, but it is also implicated in different human systemic diseases, such as infective endocarditis and, rarely, in brain abscesses, osteomyelitis, or low birth weight [1–4]. In particular, *Aa* virulence is strongly linked to the production of the leukotoxin (ltxA), homologous to repeats-in-toxin (RTX) family. Although not all *Aa* strains can determine an AgP, high levels of ltxA have been always detected in AgP related to *Aa* infection [5]. Clones with different leukotoxin-producing abilities have been identified, and this intraspecies diversity of *Aa* referred to specific genotypes named 652, JP2, and, more recently, Y4 [1, 5–7]. Clinically, it is one of the microorganisms responsible for both aggressive periodontitis (AgP) and chronic periodontitis (ChP). These affections are chronic inflammations of the periodontal tissues related to the presence of subgingival bacteria that occur in severe forms in 5–20% of adult populations, according to what was recently reported by The World Health Organization [8–10].

It has been recognized that both regular supportive periodontal therapy and the domiciliary oral hygiene, related to supragingival plaque control through a host modulation of periodontal bacteria, are essential for obtaining a good clinical outcome [11]. In this context, the use of a local chemical antiseptic able to inhibit the plaque biofilm formation could be fundamental to support the mechanical therapy [11–13]. At the same time, as previously stated, *Aa* plays an important role as an etiological agent of systemic diseases when it disseminates in the bloodstream. Oral bacteria could promote a transient bacteraemia, during all the dental procedures, with variable severity. This variability could be related to both the kind of treatment and the level of communication established between oral cavity and bloodstream [14].

In this study, three commercially available mouthwashes as DayCare, Listerine, and Dentosan have been tested. DayCare (Curaden Healthcare, Via G. Parini, 19-Saronno VA) and Listerine (Johnson & Johnson Consumer Healthcare, Morris Plains, NJ, USA) are both based on the same essential oils combination (menthol 0,042%, thymol 0,064%, methyl salicylate 0,06%, and eucalyptol 0,0092%) but differ in terms of the absence of alcohol (Daycare) or the presence of alcohol with a concentration of 26,2% (Listerine). They show both bacteriostatic and bactericidal activities that are related to cell wall disruption and enzymatic inhibition. Dentosan (Johnson & Johnson Consumer Healthcare, Morris Plains, NJ, USA) is a chlorhexidine (CHX-) based mouthrinse, and it is recognised as the most effective chemical agent, usable in the oral cavity, for biofilm inhibition. It exists in three different concentrations (0.20%, 0.12%, and 0.05%) related to its clinical use. For this analysis, the percentage of CHX chosen is 0.12%, that is, the concentration commonly used for biofilm modulation in long-term therapies [12, 15, 16]. Its antibacterial activity is linked to the capability of binding to bacterial cell membrane. This determines an increased permeability at low concentration while, at high concentration, it causes a precipitation of bacterial cytoplasm with bactericidal effect [15, 16]. 

The aim of this work is to evaluate these commercial mouthwashes activity in order to prevent *Aa* biofilm formation. In particular, their antimicrobial effect has been tested by calculating the minimum inhibitory concentration (MIC), the minimum bactericidal concentration (MBC), and the minimal eradication biofilm concentration (MEBC) [17]. Additionally, the bactericidal effect of tested mouthwashes will be evaluated in relation to the exposure time. Therefore, *Aa* viability will be estimated after 10, 20, 30, 40, 50, and 60 seconds of exposure to each mouthwashes at the MBC dilution.

The results will allow to estimate the effect on planktonic growth (PG) and on biofilm development (BD) of essential oil-based mouthwashes with or without alcohol, and it will be compared with CHX activity.

## 2. Materials and Methods

### 2.1. Bacterial Strain and Growth Condition

This study was performed using a strain of *Aggregatibacter actinomycetemcomitans* DSM 11123 [18] from the Deutsche Sammlung von Mikroorganismen und Zellkulturen GmbH (German Collection of Microorganisms and Cell Cultures). The bacteria were grown in anaerobic difficile agar (Microbiol, UTA, Cagliari, Italy) at 37°C for 24 hours with a CO_2_ concentration of 5%. After the incubation, the *Aa* was suspended in vials containing Schaedler broth to obtain a concentration with a turbidity equivalent to the No. 3 McFarland standard (about 10^8^ CFU/mL) then diluted to 1/100 (obtaining a 10^6^ CFU/mL) using a spectrophotometer at 620 nm (DMS100s, Varian, NH, USA) [19].

### 2.2. MIC, MBC, and MEBC Evaluation

To analyze antibacterial activity, 200 *μ*L of each previously described suspended strain was added to eleven wells of a 96-well plate and incubated for 48 h incubation at 37°C with a CO_2_ concentration of 5%. Before incubation, each mouthwashe was added to the bacterial suspension following a scalar concentration pair to *D* = 1/2^*n*^ (where *D* is the dilution factor and *n* is equal to a number from 1 to 11 following wells sequence). After incubation, the PG was evaluated with spectrophotometric method using a microtiter plate reader with a lecture at 620 nm (SLT-Spectra II, SLT Instruments, Germany). In order to establish when the mouthwashes reach the bactericidal concentration, whenever the spectrophotometric analysis showed an absorbance value equal to 0.000 for PG (equivalent to the absence of bacterial growth), subcultures in difficult anaerobic agar were performed to determine the MIC and MBC. To determine the MEBC, the method of the microtiter plate biofilm production assay was used [20]. All the analyses were performed three times in order to be able to do an analysis of the variance (ANOVA).

### 2.3. Exposure Time Analysis

Once the MBC was identified, the time required for each mouthwash to reach the bactericidal effect was tested. Each product was therefore placed in 6 tubes containing a bacteria concentration equal to 10^6^ CFU/mL. Sequentially, a sample was performed from each tube every 10 seconds, and a subculture in anaerobic difficile agar was prepared in order to determine the residual bacterial viability in relation with six different exposure times (from 10 to 60 seconds).

### 2.4. Microtiter Plate Biofilm Production Assay

To perform BD analysis, the method of the microtiter plate biofilm production assay was used [20, 21]. After the incubation, the medium was removed and the microtiter plate wells were washed three times, with 200 *μ*L of PBS (0.1 M, pH 7.4) buffer using a pipette, and allowed to dry for 15 min. The microtiter wells were stained with 200 *μ*L of 0.4% crystal violet for 15 min. at room temperature. The unbound crystal violet stain was removed, and the wells were washed three times with 200 *μ*L of PBS buffer. The wells were air-dried for 15 min, and the crystal violet in each well was solubilized by adding 200 *μ*L of 33% acetic acid. At the end of the coloration, the biofilm was evaluated using the microtiter plate reader (SLT-Spectra II, SLT Instruments, Germany) at 620 nm. 

### 2.5. Statistical Analysis

To analyze the different effect that each mouthwash had on BD and PG, a statistical comparison has been applied. For this reason, the absorbance values were replaced with comparing values from 0 to 1 and, in particular, the value 1 was always correlated to the absorbance value obtained with the dilution equal to 1/2^11^. To perform the comparison between different mouthwashes effect, antiplanktonic and antibiofilm activities were analyzed separately using the raw absorbance values. To compare the results obtained from different moutwashes and from different bacterial state (Planktonic or Biofilm), the absorbance raw values were converted. To perform this conversion each value were transformed into a number from 0 to 1 using as 1 the absorbance value related to the dilution equal to 1/2^11^ (i.e., if the 1/2^11^ABS raw value is equal to 1.29 it become 1, so all the previous raw value (*R*) related to the same mouthwash and the same bacterial state will be converted in this way: *x* = *R*/1.29). Statistical analysis was performed using the two-way ANOVA procedure with a dedicated software (Minitab, version 15.1.1.0 for Windows, Minitab Inc., State College, PA, USA). Statistical significance was assumed at *P* value inferior or equal to 0,05.

## 3. Results

From the statistical analysis, it was possible to highlight how bacterial viability is always related to the dilution factor (*P* < 0,0005). It is noticed that Dentosan is the most effective mouthwash against both PG and BD without any specific activity (*P*: 0,192). Listerine inhibits the BD at a minor dilution despite the dilution necessary to have the antiplanktonic activity, obtaining MEBC at the left of the MBC/MIC (1/2^4^ against 1/2^3^). However, a statistic relevance that highlighted a difference between its antiplanktonic and the antibiofilm effects was not found (*P*: 0,095) (Table 1). 

BD and PG inhibition by DayCare shows a peculiar behavior, whereas Dentosan and Listerine have only one dilution exponent of difference between MEBC and MBC/MIC (resp., 1/2^8^ against 1/2^9^ for Dentosan and 1/2^4^ against 1/2^3^ for Listerine) for DayCare two exponents of difference (1/2^4^ against 1/2^6^) with a *P*-value statistically relevant could be noticed (*P* value: 0,032) (Table 1). 

**Figure 1:**
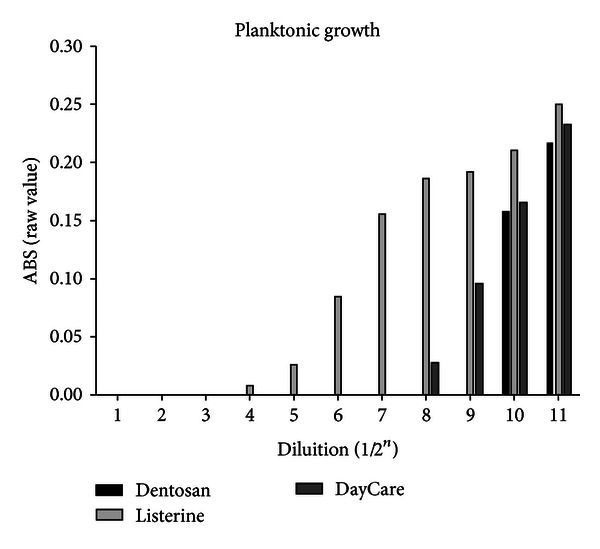
Comparison between absolute values of absorbance related to the PG of *Aa* when exposed to growing dilution (from 1/2 to 1/2048) of the tested mouthwashes.

**Figure 2:**
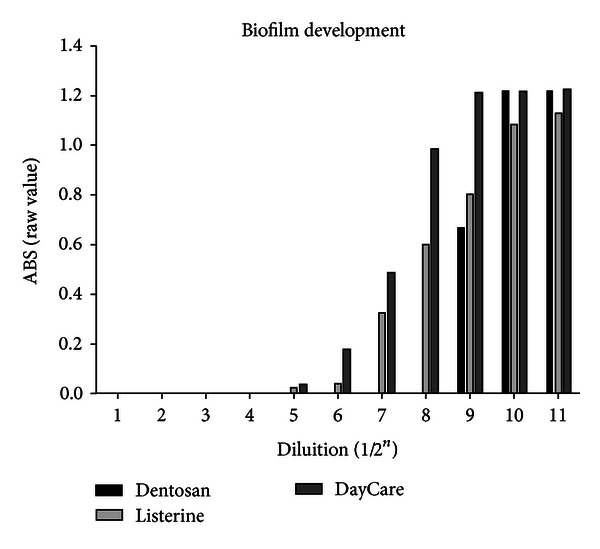
Comparison between absolute values of absorbance related to the BD of *Aa* when exposed to growing dilution (from 1/2 to 1/2048) of the tested mouthwashes.

By the analysis of antiplanktonic and antibiofilm activities separately, it was possible to determine some differences between mouthwashes in terms of raw ABS values. In both of the antibacterial effects, there is a statistical difference related to the mouthwash examined (*P*: 0,002 for anti-planktonic effect and *P*: 0,017 for antibiofilm activity). For MBC/MIC activity, the worst mouthwash is Listerine with an optically determined bacterial growth, yet with a concentration of 1/2^4^, followed by DayCare at 1/2^8^. The best is Dentosan that inhibited bacterial growth until a dilution of 1/2^9^ (Table 1). In the antibiofilm evaluation, Listerine was the first mouthwash in which it was possible to measure the BD with an MEBC at a dilution of 1/2^4^. Although the Listerine does not have a high MEBC, it showed to have a good biofilm inhibition until the higher dilution tested (1/2^11^) with ABS values minor then registered for Dentosan. As for the MBEC, the best activity was again the one of Dentosan (1/2^8^) while the worst effect was found for DayCare that showed the highest level of ABS for biofilm, yet with a dilution of 1/2^7^ (Figure 2, Table 1). In analyzing the raw values of absorbance related to PG and BD separately it was possible to highlight a good activity of DayCare against PG in contrast to the worst activity on BD inhibition related to other mouthwashes (Table 1, Figures 1 and 2). All mouthwashes tested presented a good antibacterial activity when they are used at concentration under a dilution of 1/2^4^. From the exposure time analysis, Dentosan and Listerine were found to inhibit bacterial viability after only 10 and 20 seconds, respectively, while DayCare needs 30 seconds to obtain a totally bactericidal effect. 

## 4. Discussion

Recently, mouthwashes have obtained a great importance as therapeutic keystone against oral infections such as caries, candidiasis, and especially periodontitis. Their activity can be applied for pathology prevention, as well as for preventing systemic pathologies linked to transient bacteraemia that is always generated during dental manipulations [3, 14, 22]. A good mouthwash should have a good antibacterial activity against both PG and BD in order to prevent all these conditions. The present analysis is focused on the study of this double effect, fundamental to evaluate the real antibacterial power of each mouthwash [23].

From the results, the huge difference between mouthwashes activity against *Aa* in its planktonic and biofilm forms clearly appears. Firstly, alcoholic products behave in a different way from the other two mouthwashes so that, while they have the worst effect against PG, they show at the same time the best antibiofilm activity at high dilution. In accordance with the literature, essential oils with alcohol have the best antibiofilm activity [23–25]. On the base of these results, it seems that an alcoholic mouthwash could be a good choice in preventing plaque formation after periodontal therapies. 

In contrast, essential oil nonalcoholic mouthwashes show a better antiplanktonic rather than antibiofilm activity. In this case, it could be considered a good choice in order to prevent bacterial systemic dissemination. In particular, they could be useful for treating specific anatomic areas with a high risk of bacteraemia. 

At the same time, it is reported how transient bacteraemia could be determined not only during some clinical procedures or in particular conditions but also through subclinical dental infection or daily dental brushing [14, 26]. Patients with underlying cardiopathy or with oral condition with high risk of oral bacteria dissemination need to be supported by an adequate prophylaxis through local antiseptic products. Although *Aa* PG seems to be susceptible to all tested mouthwashes, it becomes necessary to refer to a mouthwash with a strong anti-planktonic activity in case of therapies with an high risk of bacterial dissemination from the oral cavity. 

In conclusion, this work acknowledges how both essential oil-based mouthwashes, with or without alcohol, have a good and comparable antibacterial power when they are used pure on *Aa*. In accordance with the literature, it was demonstrated that all of them showed the bactericidal effect within the maximum exposure time *in vivo* (60 seconds) even though the bacterial response fluctuates significantly after consecutive dilutions of tested antiseptics, as previously described. However, further studies on the chemical impact of alcohol as well as the action of the active ingredients of the referred mouthwashes will be needed to more full investigation. 

These results suggest that it is necessary for clinicians to understand both mouthwashes molecules and the pathogenic agents involved in order to obtain a therapeutic effect. Further studies will be needed to confirm these results *in vivo *and on others bacterial species.

## Figures and Tables

**Table 1:** The table is divided to three parts: in the first part the statistical analysis of total antibacterial effect by using of absolute values related to PG and PD separately is shown. In the second part there is the statistical analysis of anti-PG and anti-BD activity separately. In the third part MIC, MBC, and MEBC for each mouthwash are shown.

	Sup.	Mean	Inf.	Dv.St	PG mean	BD mean	Dev.St	*P* value	MBC	MIC	MBEC
Dentosan	0,340	0,182	0,024	0,356	0,134	0,231	0,069	0,192	1/2^9^	1/2^9^	1/2^8^
Listerine	**0,512**	**0,345**	**0,178**	0,376	**0,396**	0,297	0,070	0,095	1/2^3^	1/2^3^	1/2^4^
DayCare	0,468	0,291	0,114	**0,398**	0,186	**0,396**	0,149	**0,032**	1/2^6^	1/2^6^	1/2^4^
